# Case of Pyæmia after Amputation

**Published:** 1867-05

**Authors:** E. R. Barnes


ART II—*Case of Pyæmia after Amputation.* By E. R. Barnes, M. D.

J. L., aged 56, desired amputation of left leg, for removal of deformity called talipes equineus.

History.—The deformity was observed at the age of two years; the patient having previously suffered from convulsions. The general health of the patient through life had been good. He had been accustomed to the free use of malt liquors. As the patient advanced in years the difficulty of walking continually increased. The weight of the body being thrown upon the extremities of the metatarsal bones, the subjacent parts became tender, and finally the seat of ulcerations which rendered locomotion very painful.

Appearance of Limb.—The foot was extended in nearly a straight line with the leg, and much shortened. The flexor muscles of the left forearm were also contracted, producing partial closure of the hand. The left arm and leg were atrophied in a degree corresponding to the diminished use of the muscles.

Tuesday, January 22d, 1867.—The patient having been prepared as usual, Dr. Miner removed the leg at the junction of the middle and lower third, by the flap operation. The limb was placed on a pillow and the dressings were kept moist with tepid water. Contractions of the flexor muscles of the thigh ensued within a half hour, more severe than usual. A teaspoonful of McMunn's Elixir Opii was ordered, to be repeated every four hours if indicated.

Jan'y 23d.—The contractions had so much increased as, at brief intervals, to forcibly and completely flex the leg on the thigh and the thigh on the trunk, requiring unremitting attention to prevent injury to the stump. A double inclined plane was therefore firmly secured to the bed, and thickly padded with folds of flannel of sufficient width to embrace the sides of the limb. A roller bandage, passed under the splint and over the thigh and leg, controlled the, movements and seemed to give much relief to the minds of both patient and friends. Injected hypodermically, one-half grain of muriate of morphia.

Jan'y 24th.—The hypodermic injection only temporarily checked the frequency and force of the contractions. On removing dressings, found the swelling moderate, and observed a slight dusky hue along the inner edge of anterior flap, and extending up the inner border of the tibia about four inches. Considerable febrile reaction; pulse 110; countenance anxious; tongue natural. Continued anodyne.

Jan'y 25th.—Pulse 120; expression anxious; face bathed in perspiration; contractions frequent and severe, with forcible opening and closing of jaws. As the left extremity could not yield, the movement appeared on the right side. Dusky hue, well marked, with some redness.

Heretofore the patient had been kept fully under influence of anodyne, and had taken a tablespoonful of whisky at intervals. Having been accustomed to the use of beer this was substituted, and the anodyne was discontinued, except one dose at night. Slight, thin, dark discharge from stump.

Jan'y 26th.—General appearance better; pulse 100; discharge more free, but thin and discolored. Erysipelas had distinctly appeared in anterior flap, and region previously discolored.

Jan'y 27th.—Improving in general; erysipelas spreading. Ordered enema, which caused moderate evacuation. Used warm water dressings; removed two sutures.

Jan'y 28th.—Bowels moved freely. Erysipelas had extended up to knee, covering leg anteriorly, with much swelling and tension. Posteriorly, leg quite natural. Took out all sutures; removed splint; endeavored to maintain partial extension by suspending limb.

Jan'y 29th.—Erysipelas had not extended. Discharge abundant, and in color and consistence better, but not laudable. Odor offensive, requiring all dressings to be removed, twice a day.—Placed limb upon a pillow, flexed, but at second visit found he had soon cast this off. Renewed suspension.

Jan'y 30th.—The flexor muscles of the thigh having maintained powerful traction on the leg, causing it to bear firmly upon the sling used for suspension, had produced some congestion of flaps. Further efforts to maintain extension were abandoned. The leg was allowed to assume a position of complete flexion on the thigh. A pillow was placed under the limb, securely fastened to both thigh and leg, and supported so as to protect the stump from injury. In this position the limb subsequently remained.

Jan'y 31st.—Pulse 85; skin and tongue natural; appetite good; erysipelas had ceased to extend; warm water dressings discontinued; used simple cerate on dressings, next to skin; narrow slough on edge of anterior flap; discharge free, but dark and offensive.

Feb'y 1st.—Copious purulent discharge, slightly greenish.

Feb'y 2d.—Removed some sloughs of cellular tissue; erysipelas much diminished; general condition good; pulse 75.

Feb'y 7th.—Removed ligatures; discharge laudable; erysipelas nearly or quite disappeared; general condition good. Patient complained of pain along inner border of tibia, most acute near its extremity, which he had felt for many days.

Feb'y 15th.—To date, the case had apparently progressed favorably. The erysipelatous inflammation disappeared, leaving however a point near the extremity of the tibia excessively painful to the touch. Skin over this point was bluish, thin, and sank into a circular depression three-fourths of an inch in diameter. Pressure on this point caused a thin discharge from edge of flaps. Flaps entirely united except two small openings. A probe passed through these reached the sawn end of tibia, which was rough. The only discharge which remained, came through these openings from the spot indicated. In all other respects stump seemed perfectly healthy. A small abscess of the cellular tissue appeared behind the left tuber ischii, which quickly healed on removing pressure. Patient had complained for three or four days of having caught cold, which caused a slight cough and some hoarseness at times.

On the night of the 14th February he was attacked with pain in the right side, aggravated by a full inspiration, very acute on coughing. Patient's condition was much changed at date. Temper irritable; expression somewhat anxious; pulse 100, and weaker; tongue coated; appetite diminished; sputa tenacious, and raised with difficulty; no dullness observed on percussion; crepitant rales. Patient had voluntarily applied mustard to chest, from which, he said, was some alleviation of pain. As bowels had not moved for two days, ordered an enema. Gave a mild expectorant and disphoretic.

February 15. Pulse 105; sputa streaked with blood; urine high colored; complained now of pain in right lumbar region; sat up several hours; in moving, said right leg was getting weak; when reclining, said it was the seat of slight, dull pain; enema did not act; moved bowels by ol. ricini; taking stimulants and nourishing food.

Feb. 17. No change.

Feb. 18. The right lower extremity exhibited a diminished temperature and a languid circulation; the skin presented a mottled appearance; had pain throughout limb; desired it supported under knee by pillow; no morbid appearance about stump or left lower extremity; ordered warm applications to whole of right limb.

Feb. 19. During twenty-four hours the right lower extremity had immensely swollen; skin tense; tissues hard; color dark and purplish; temperature unchanged; painful; thigh very painful to the touch; patient said he felt now no pain in chest or abdomen; pulse rapid and very weak; countenance very anxious, shrunken and bathed in perspiration. An incision deep into the thigh was followed by dark, sluggish blood. Stump diminished in size and much whiter than before; cicatrix blueish; no morbid appearance about left limb; applied fomentations to right limb and ordered stimulants every half hour.

Feb. 20. No vitality remained in right leg; patient died at six P. M.

The cause of death in this case was, apparently, that vitiated condition of the blood denominated pyaemia, which first manifested itself in the access of pneumonia in the right lung, with the decided constitutional change then noted, and which caused phlebitis, subsequently developed throughout the right lower extremity. The leg had, anteriorly, been the seat of erysipelas, which on subsiding left a point near the extremity of the tibia, the seat of a low form of inflammation. This existed at the time of the occurrence of pyaemia. During the progress of this case I was several times assisted by the counsel of Dr. Miner.

On dissecting the bones of the foot, it was seen that the phalanges were at right angles to the metatarsal bones; the plantar ligaments were much shortened, drawing downwards the os-calcis; the head of the astragalus presented a large, well-rounded articular surface; the body of the astragalus was tilted inwards, leaving a space between its outer margin and that of the os-calcis of more than half an inch. In this space had grown up, from the astragalus and from the os-calcis each, a new osseous growth, terminating in a facet, that of the astragalus looking backward, that of the os-calcis looking forward. These moved upon each other as do the articulating facets of the spinal vertabræ. The surface of the astragalus, intended for articulation with the tibia and fibula, was eroded, while the bones of the leg had formed a new articulating surface, extending back half an inch on to the os-calcis. Into this new joint extended from behind, two wedge-shaped bones, one symmetrical, the other irregular.

The motions of the foot were two in number, and very limited. That of flexion and extension, was between the surfaces of the tibia and fibula, and astragalus and os-calcis. The lateral motion was produced by the play of the astragalus, through its head and facet upon the scaphoid and os-calcis.

